# Rebuilding the Strain Hardening at a Large Strain in Twinned Au Nanowires

**DOI:** 10.3390/nano8100848

**Published:** 2018-10-18

**Authors:** Jiapeng Sun, Jing Han, Zhenquan Yang, Huan Liu, Dan Song, Aibin Ma, Liang Fang

**Affiliations:** 1 College of Mechanics and Materials, Hohai University, Nanjing 210098, China; yang-zhen-quan@hotmail.com (Z.Y.); liuhuanseu@hhu.edu.cn (H.L.); songdancharls@hhu.edu.cn (D.S.); aibin-ma@hhu.edu.cn (A.M.); 2 School of Mechanical and Electrical Engineering, China University of Mining and Technology, Xuzhou 221116, China; hanjingcumt@gmail.com; 3 State Key Laboratory for Mechanical Behavior of Materials, Xi’an Jiaotong University, Xi’an 710049, China; fangl@xjtu.edu.cn

**Keywords:** twinned, nanowires, strain hardening

## Abstract

Metallic nanowires usually exhibit ultrahigh strength but low tensile ductility, owing to their limited strain hardening capability. Here, our larger scale molecular dynamics simulations demonstrated that we could rebuild the highly desirable strain hardening behavior at a large strain (0.21 to 0.31) in twinned Au nanowires by changing twin orientation, which strongly contrasts with the strain hardening at the incipient plastic deformation in low stacking-fault energy metals nanowires. Because of this strain hardening, an improved ductility is achieved. With the change of twin orientation, a competing effect between partial dislocation propagation and twin migration is observed in nanowires with slant twin boundaries. When twin migration gains the upper hand, the strain hardening occurs. Otherwise, the strain softening occurs. As the twin orientation increases from 0° to 90°, the dominating deformation mechanism shifts from slip-twin boundary interaction to dislocation slip, twin migration, and slip transmission in sequence. Our work could not only deepen our understanding of the mechanical behavior and deformation mechanism of twinned Au nanowires, but also provide new insights into enhancing the strength and ductility of nanowires by engineering the nanoscale twins.

## 1. Introduction

Metallic nanowires have drawn a great of interest and have been extensively studied in recent years because of their unique properties and potential as a fundamental building block of nanotechnology. Many nanomechanical experiments and molecular dynamics (MD) simulations have proved that metal nanowires possess extreme-high strength (Au, Ag, Cu, Ni, Al), and exhibit “smaller is stronger” trend [1,2,3,4]. However, although such nanowires fail at higher stresses, they may exhibit either limited or no strain hardening capability at all. For example, the whisker-like mechanical behavior, in which the stress strain curves indicate a sharp yield point, followed by severe strain-softening, is generally observed in the deformation of single crystalline metal nanowires, where dislocation or deformation twinning or lattice reorientation sustains the plastic deformation [5,6,7,8].

The strain hardening behavior is highly desirable, as it serves to delay the onset of necking, thus promoting tensile ductility. To promote strain hardening, barriers to dislocation motion should be drew into nanowires. One can engineer grain boundaries into nanowire, i.e., nanocrystalline nanowires. The general grain boundaries could rebuild the strain hardening capability but decrease the yield stress [9,10,11,12]. An attractive strategy is engineering coherent twin boundaries (CTBs) with nanometer-scale spacing into nanowires, i.e., nanotwinned nanowires (NT nanowires). Several NT nanowires are founded to exhibit extreme-high strength and even ideal strength, such as NT Cu [13,14,15], Ni [16,17], Au [8,18,19], Ag [20,21], and Al [21]. Interestingly, the desired strain hardening capability can be rebuilt in low stacking-fault energy metals nanowires, such as NT Au and NT Ag nanowires containing orthogonally oriented CTBs [21,22] and fivefold vertical CTBs [22]. In contrast, no strain hardening can be found in NT nanowires with high stacking-fault energy, like NT Cu and NT Ni nanowires. This fundamental difference is attributable to the relative change between the stress required to nucleate new dislocations from free surfaces and that for the dislocation transmission at CTBs as a function of the unstable stacking-fault energy [21,22].

NT Cu and Au nanowires containing orthogonally oriented CTBs have been fabricated successfully [13,18], and thus their mechanical behavior and deformation mechanism were widely investigated in experiments and MD simulations. Jang et al. [13] revealed that the strength of NT Cu nanopillar is up to 2.5 GPa, as about 1.5 times higher as the single crystal counterpart using an advanced an in-situ tension experiment and MD simulation. The ductile-to-brittle transition as the twin spacing decreasing was observed for the nanopillars with a diameter of 100 nm. Similar ultrahigh strength (3.12 GPa, near the ideal limit of Au) and the ductile-to-brittle transition were reported in the NT Au nanowires by Wang et al. [9]. The MD simulations revealed that the CTBs could cause strengthening effects, weakening effects, and no effects, as a function of both twin spacing and sample diameter [15,23,24]. Special dislocation-CTBs interactions are found to be responsible for the special mechanical behavior of NT nanowires. It has been generally accepted that the incipient plastic deformation of NT nanowires proceeds via the nucleation and emission of partials from specific sites where CTBs intersect planes on the free surface, which is very different from the twin free nanowires. Hence, the additional repulsive force that is exerted by CTBs on the dislocation nucleation is introduced to be responsible for the observed ultrahigh strength [25]. In fact, CTBs not only can sever as a generator of the repulsive force which acts against the nucleation of dislocations, but also as a source of dislocation nucleation [24,26]. By investigating the tensile deformation of NT Au nanowires depending on the twin spacing and length, Guo et al. [24] given a further explanation on size dependent strength using competing mechanisms in the initial yield, dislocation source number vs. repulsive force. Furthermore, different dislocation-CTBs interactions, such as cross-slipping, transmission, and reaction were observed when a sliding dislocation encounters a CTBs. The detailed and comprehensive description can be found in Zhu’s review paper [27].

Recently, some attention is paid on the NT nanowires containing slant and parallel CTBs. The slant CTBs are subjected to nonzero resolved shear stresses during axial tension/compression of nanowires. Thus, twin migration or detwinning is a governing deformation mechanism, and thus facilitates a large amount of plasticity [13,28,29]. Experiments and MD simulations all proved that the mechanical behavior and deformation mechanism were significantly related to the twin orientation respective to the load direction. Lu et al. [30] explored the correlation between twin orientation, active slip systems, dislocation structure, and dislocation interaction during the compression of highly oriented NT Cu in a transmission electron microscope (TEM). Three slip modes and different interactions of dislocations were observed in deformation at 0°, 45°, and 90° with respect to CTBs. The detailed dislocation process and mechanical behavior of NT Cu nanowires were examined by recent MD simulations [29,31,32]. The results demonstrated that the dominant deformation mechanism of NT Cu nanowires transits dynamically from slip transmission to twin boundary migration to slip-twin interactions as the twin boundary orientation changes from horizontal to slant, and then to a vertical direction.

The existing experiments and MD simulations showed that the mechanical behavior and deformation mechanism of NT nanowires were not only related to twin spacing and sample size, but also significantly related to the angle between loading axis and CTBs. Thus, a desirable combination of high strength, ductility, and strain hardening can be achieved by proper arrangement of twin orientation. However, systematic research on twin orientation and twin spacing dependent mechanical behavior and deformation mechanism was seldom involved so far, especially for the NT nanowires beyond Cu. The strain hardening of NT nanowires as a function of twin orientation and twin spacing is still an open issue, which are crucial in advancing the understanding of plastic anisotropy of NT nanowires, and are helpful to develop the polycrystalline NT metals with randomly oriented CTBs.

Here, our larger scale molecular dynamics simulations demonstrated that we could rebuild the highly desirable strain hardening behavior at a large strain in twinned Au nanowires by changing twin orientation, which strongly contrasts with the strain hardening at incipient plastic deformation in low stacking-fault energy metals nanowires. Because of this unique strain hardening, improved ductility is achieved. The detailed dislocation process during plastic deformation and detailed dislocation-CTBs interactions were identified to clarify the deformation mechanism at the atomic level.

## 2. Materials and Methods

**Figure 1:**
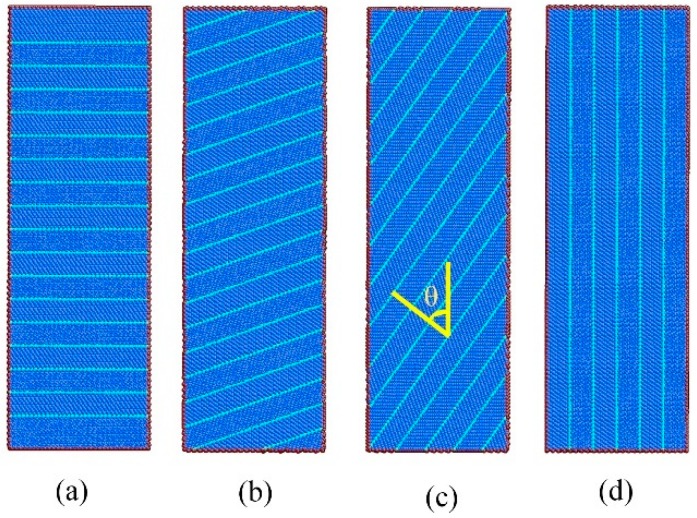
Cross-section view of the nanotwinned (NT) Au nanowires with (**a**) *θ* = 0°, (**b**) *θ* = 20°, (**c**) *θ* = 54°, and (**d**) *θ* = 90°. The atoms are colored according to the common neighbor analysis (CNA). The blue, sky blue, and red atoms are FCC, HCP, and other atoms.

Large-scale molecular dynamics simulations were performed with an embedded atom-method potential that was developed by Grochola et al. [33]. This potential accurately predicts the surface energy and the stacking fault energy of gold (i.e., 43.4 mJ·m^−2^, which is in reasonable agreement with the experimental data 32–46.4 mJ·m^−2^). The cylindrical NT nanowires were constructed with a constant initial diameter of 17 nm and initial length of 51 nm, which means a length-diameter ratio of 3. The nanowires consisted of ~720,000 atoms. The twin orientation angle θ (signified by the angle between the load axis and the normal of CTBs) was chosen 0°, 19.47°, 54.74°, and 90°, as illustrated in Figure 1. The slant CTBs were achieved by rotating the CTBs along [−110] axial to the specific angles. Four specific angles were chosen to maintain the load axis along the lattice orientation with low indices, as listed in Table 1. The twin spacing λ (i.e., the spacing between adjacent CTBs) was chosen to 1.41 nm, 2.83 nm, and 5.45 nm. The free boundary conditions were imposed along all directions, indicating the finite length nanowires. The atoms in a layer with thickness of 1 nm at one end of the nanowires were frozen to provide structural stability, and similar atoms at the other end were frozen to impose the load.

After the initial construction, a meticulous heat treatment process was performed to obtain the equilibrated construction at a temperature of 300 K [14,19]. The initial nanowires were firstly heated from 0 K to 300 K over 0.25 ns, and then annealed at 300 K for 2.5 ns under the NVT ensemble using a Nosé–Hoover temperature thermostat. Then, nanowires underwent a uniaxial tension at a constant strain rate of 5 × 10^7^ s^−1^ and a temperature of 300 K. The typical duration for tension simulation were 15 ns. To capture the defect evolution and identify dislocations, the atoms are colored according to common neighbor analysis (CNA) [34] and slip vector analysis (SVA) [35]. All of the simulations were performed utilizing the Verlet integration algorithm with a time step of 5 fs by large-scale atomic/molecular massively parallel simulator (LAMMPS) [36].

## 3. Results and Discussion

### 3.1. Mechanical Behavior of the NT Au Nanowires at Small Strain

**Figure 2:**
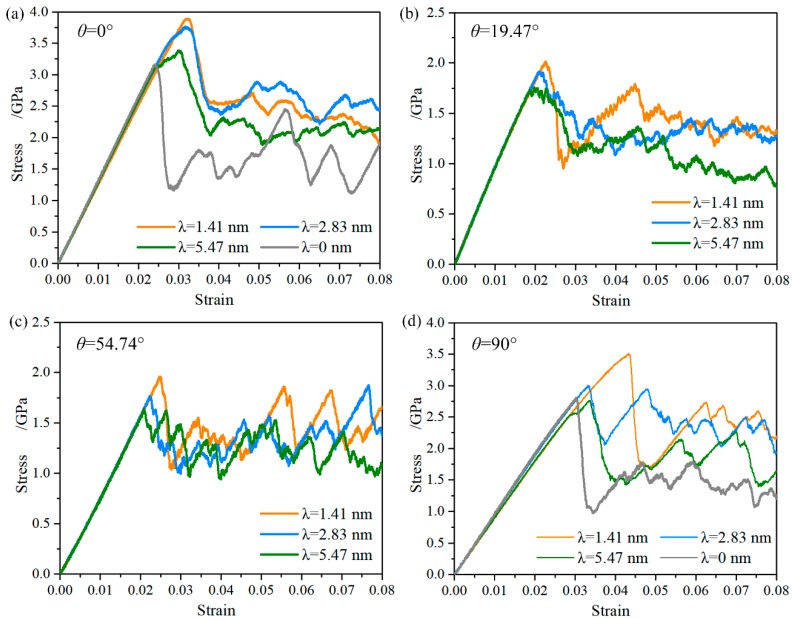
Engineering stress-strain curves of the NT Au nanowires with (**a**) θ = 0°, (**b**) θ = 20°, (**c**) θ = 54°, and (**d**) θ = 90° in the strain range of 0 to 0.05.

Figure 2 shows the stress-strain curves of the NT Au nanowires with different twin orientation and spacing in a small strain range of 0 to 0.08. For comparison, the stress-strain curve of the <111> oriented single crystal nanowire (i.e., the NT Au nanowires with λ = 0), which has same orientation of load axis to the NT Au nanowires with θ = 0°, is also shown in this Figure, as well as the <112> oriented single crystal nanowires. Obviously, the mechanical response of NT Au nanowires significantly depends on the twin orientation. A pronounced shift in mechanical behavior from whisker-like deformation to strain-hardened regime is found as the twin orientation changed from 0° and 90° to 19.47° and 54.74°. This strain hardening behavior occurred at incipient plastic deformation (before the first stress drop), which has been generally reported in low stacking-fault energy metals nanowires with orthogonally oriented CTBs [21,22]. Although the NT nanowires with θ = 0° and θ = 90° all show strain hardening, the detailed plastic flow is different. For the NT nanowires with θ = 0°, the stress linearly increases as the strain increases, followed by a well-marked yield points where the stress drops. With the increase in the twin spacing, the ultimate strength tends to the yield strength, indicating wakening strain hardening, as shown in Figure 2a. In contrast, the NT nanowires with θ = 90° show smooth elastic-plastic transition where the stress linearly increases, followed by sublinear increasing with the increase of applied strain when the twin spacing is below 5.45 nm. This specific hardening behavior was also observed in the five-fold NT Ag nanowires [22]. Moreover, the obvious strengthening effect by CTBs is found and enhances with the decrease in the twin spacing, as shown in Figure 2a,b, which has been widely reported and well understood in literatures [13,14,15,16,17,18,19,20,21].

**Figure 3:**
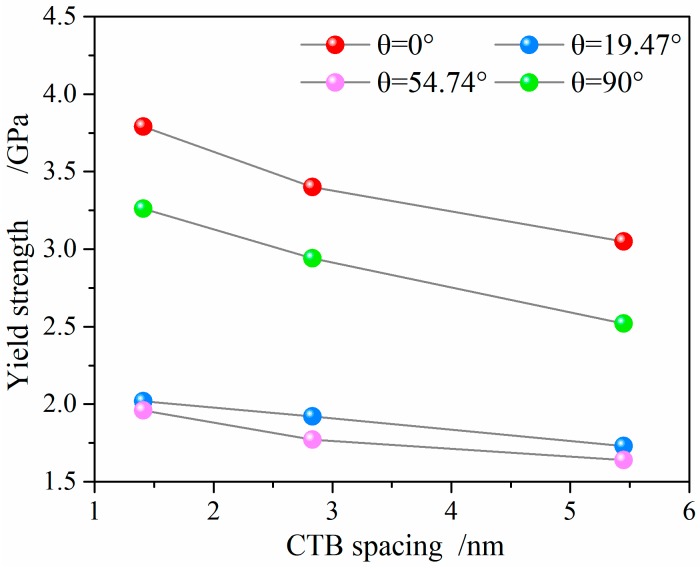
Yield strength as a function of twin orientation and spacing.

The yield strength of the NT Au nanowires, which is defined by the nucleation of the first dislocation, is related to both the twin orientation and the twin spacing, as shown in Figure 3. The NT Au nanowires with four different twin orientation all have increased yield strength as the twin spacing decreases. The NT Au nanowire with *θ* = 0° and λ = 1.41 nm has a maximum yield strength of 4.04 GPa and maximum ultimate strength of 4.14 GPa. In contrast, the NT Au nanowires with *θ* = 54.74° and λ = 5.45 nm has a minimum yield strength of 1.89 GPa. The yield strength of the NT nanowires with *θ* = 90° is slightly smaller than those with *θ* = 0°, but much larger than those with *θ* = 19.47° and *θ* = 54.74°. This result indicates that the orthogonally oriented CTBs have an optimal strengthening effect, followed by the parallel CTBs, and slant CTBs is least in the NT Au nanowires.

### 3.2. Mechanical Behavior of the NT Au Nanowires with θ = 54.74°

**Figure 4:**
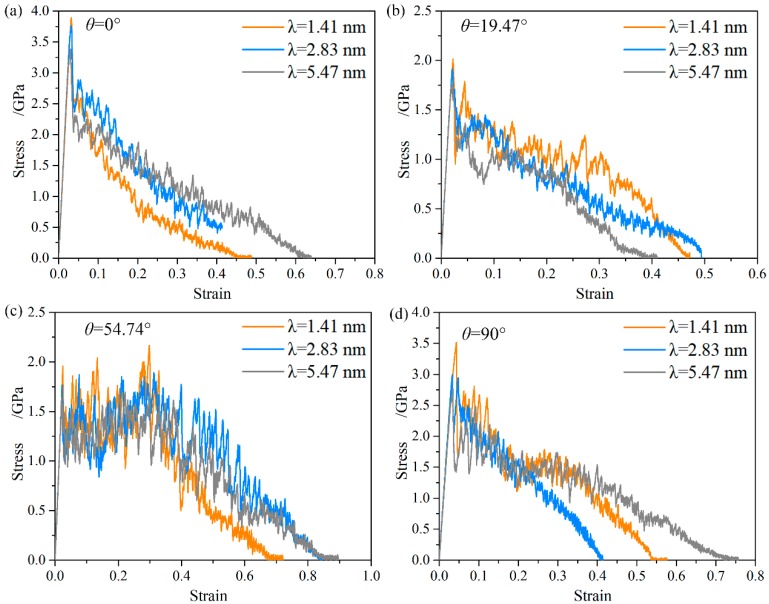
Engineering stress-strain curves of the NT Au nanowires with different twin orientation and twin spacing. (**a**) θ = 0°, (**b**) θ = 19.47°, (**c**) θ = 54.74°, and(**d**) θ = 90°.

Figure 4 shows the unabridged stress-strain curves of the NT Au nanowires with different twin orientation. A unique deformation behavior and strain hardening behavior are observed in the NT Au nanowires with θ = 54.47°. For this type of NT nanowires, the serrated stress increases to its maximum in a large strain range of 0.21 to 0.31, which is far away from the yield point, and then drops until failure, as indicated in Figure 4c. In contrast, for the NT Au nanowires with θ = 0°, 19.47°, and 90°, the stress sharply drops beyond the ultimate strength until failure, as shown in Figure 4a,b,d, which agrees well with what was generally observed in single nanowires [4,5,17,35] and NT Cu nanowires [14,15]. It is worth noting that this unique hardening behavior in the NT Au nanowires with θ = 54.47° is very different from that in NT nanowire with θ = 0° and θ = 90° where the strain hardening occurs in the initial stage of plastic deformation and before the first stress drop.

**Figure 5:**
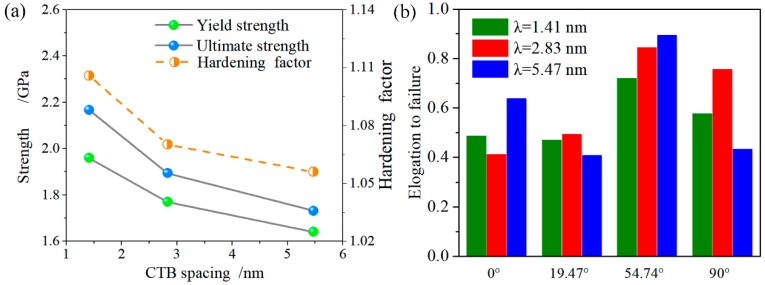
(**a**) The yield strength, ultimate strength, hardening factor as a function of twin spacing for the NT nanowires with θ = 54.74°; and, (**b**) elongation to failure versus twin orientation and spacing.

The strain-hardening factor is further used to characterize the strain hardening effect, which is the rate between the ultimate strength and the yield strength. As shown in Figure 5a, the yield strength, ultimate strength, and hardening factor all increase with the decrease of the twin spacing. In fact, the twin spacing has limited effect on the hardening factor. This result states that increasing the density of CTBs can significantly strengthen the NT Au nanowires with θ = 54.47°, accompanying with the slightly increased strain hardening capability. As this unique strain hardening behavior, the NT Au nanowires with θ = 54.47° shows improved elongation to failure, as shown in Figure 5b.

The similar strain hardening behavior has been observed in the NT Cu nanowires with square cross section and slant CTBs [29], when the oriented angle is 19.47°, 35.26°, and 54.74°. In contrast, the unique strain hardening behavior occurs only as the oriented angle is 54.74° in the NT Au nanowires. The hardening factor of the NT Cu nanowires is larger than that of the NT Au nanowires. Generally, the strain hardening behavior is highly desirable, as it means homogeneous deformation and it serves to delay the onset of necking, thus promoting tensile ductility.

### 3.3. Yielding Mechanism

**Figure 6:**
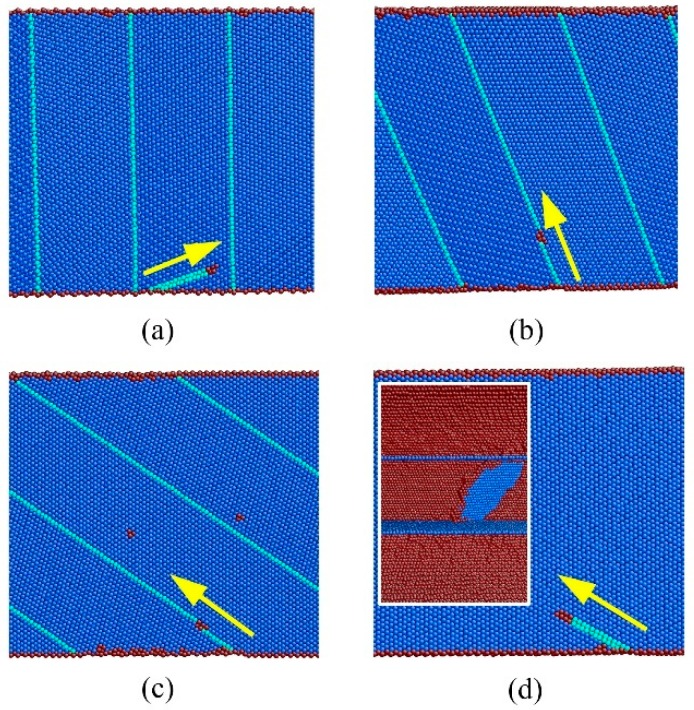
Cross-section views of atomic snapshot at yield point in NT nanowires with λ = 5.45 nm and θ = (**a**) 0°, (**b**) 19.47°, (**c**) 54.74°, and (**d**) 90 °. The atoms are colored according to the CNA. The blue, sky blue, and red atoms are atoms with FCC, HCP lattice and defective atoms, respectively. The insert in (**d**) shows the side cross-sectional view in which the atoms with FCC lattice are hid, and blue and red atoms are HCP and other atoms.

Figure 6 demonstrates atomic-scale analysis of yielding mechanism as a function of twin orientation, where the twin spacing is chosen to 5.45 nm for convenience. This Figure confirms that the onset of plasticity in all the NT Au nanowires is associated with the emission of partial dislocation from the free surface. However, three distinctive mechanisms of dislocation emission, referred to as Mechanisms I–III in the following, can be observed in the NT Au nanowires at the yield point, depending on the twin orientation.

In Mechanisms I, a 90° leading partial γD is nucleated from the CTB-surface intersection site on the free surface, and it propagates on the BCD plane, as shown on the close-up view in Figure 6a for the NT nanowires with θ = 0°. Because both the slip plane and Burgers vector are inclined to CTBs, this dislocation is immediately obstructed by the adjoining CTB. This dislocation-CTB interaction is generally referred to hard mode I [37]. As a result of the dislocation blockage, strain hardening occurs in these NT Au nanowires with further applied strain. The ultimate stress is achieved when the trailing partial emits from the stacking faults-surface intersection site on the free surface, which is left by the first leading partial. The detailed dislocation process has been generally reported in references [14,21,38].

In Mechanisms II, it is observed that a twinning partial emits from the CTB-surface intersection site on the free surface, which is generally referred to as the soft mode. This yield mechanism takes places in the NT Au nanowires with slanted CTBs (θ = 19.47° and θ = 54.74°), as illustrated in Figure 6b,c. Immediately following the nucleation, this twinning partial unrestrictedly propagates across the nanowires on the CTB, which mediates the twin boundary migration, i.e., deformation-induced twinning or detwinning.

Mechanisms III is observed in the NT nanowires with θ = 90°. In this case, a 30◦ partial αC slipped on the BCD plane associated with the formation of a stacking fault emits from the site on the surface between two adjacent CTBs, as indicated in Figure 6d. The sliding of this leading partial is restricted by CTBs, as illustrated in the insert of Figure 6d. As this restricted sliding, strain hardening is also found in these NT Au nanowires with further applied strain, as described below.

### 3.4. Strain Hardening Mechanism of the NT Nanowires with θ = 90°

The present result indicates that the NT Au nanowires with orthogonally oriented CTBs (θ = 0°) and parallel CTBs (θ = 90°) show general strain hardening in the initial stage of plastic deformation after the yield event. The detailed strain hardening mechanism has been commonly reported for the NT Au nanowires with orthogonally oriented CTBs, i.e., restricted dislocation sliding by twin boundary [21,38]. The slip-CTB interaction is mainly responsible for the plastic deformation in this nanowire. However, a detailed dislocation process for the NT Au nanowires with parallel and slant CTBs is still less clear.

**Figure 7:**
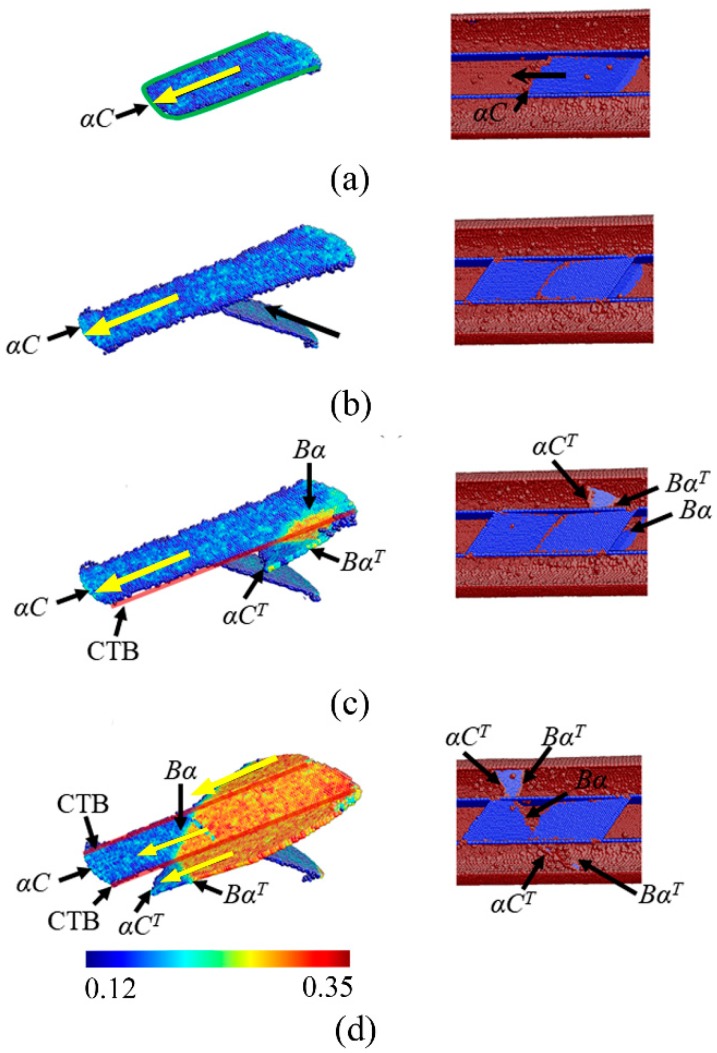
Snapshot of the NT Au nanowire with θ = 90° and λ = 5.45 nm during stretching at stain of (**a**) 2.95%, (**b**) 3.13%, (**c**) 3.38%, and (**d**) 3.43%. On the left panel, the atoms are colored according to the slip vector analysis (SVA), where the atoms with SVA less than 0.12 nm are hid. On the right panel, the atoms are colored according to the CNA, where the atoms with FCC lattice are hid. The green curve illustrates the dislocation line and the red lines mark the CTBs. The yellow arrows denote the direction of dislocation slip.

A typical example of the atomic-scale deformation in the NT Au nanowires with parallel CTBs is represented in Figure 7. Following nucleation, the 30° partial αC is immediately blocked by two adjacent CTBs along its both sides, as shown in Figure 7a. However, this partial can still slip on the BCD plane with further applied stain. Thus, a U-shape partial loop is observed in the nanowires. The straight-line segment of U-shape partial loop is blocked by the CTBs, and the arc segment slips unrestrictedly, as illustrated in Figure 7a. The U-shape partial loop continually propagates across the nanowires with increasing strain, which leaves two straight-line dislocations within the nanowires, as shown in Figure 7b. Accompanying with the propagating of the first partial loop, another partial on other slip systems emits from the free surface and slips in the similar way, which leaves a stacking fault intersected with the first stacking fault. Because of the restricted sliding of the U-shape partial loops, the significant strain hardening occurs in the NT nanowires with parallel CTBs. Obviously, this strain hardening mechanism is very different from the NT nanowires with orthogonally oriented CTBs, which is resulted by the thorough dislocation blockage [38].

The ultimate flow stress is achieved when a 30° trailing partial Bα emits following the 30◦ partial αC on the surface. The sliding of the trailing partial on BCD plane eliminates the stacking fault with further applied strain through the following dislocation reaction:

Bα + αC→BC(1)

The new resulted dislocation BC is a full dislocation. When the BC reaches the CTB, it becomes a perfect screw dislocation because the dislocation line is parallel to the Burgers vector. BC can easily transmit across the CTB (see Figure 7c), as following:

BC→BC^T^ (2)

where BC^T^ is a full dislocation that can slip on BCT^T^ plane in the twin. Subsequently, this full dislocation dissociates into two 30° partials separated by a stacking fault in the twin, i.e.,

BC^T^→Bα^T^ + αC^T^ (3)

So, an extended dislocation forms in the twin, as shown in Figure 7d. The overall dislocation reaction processes at the CTBs can be obtained by adding the reactions (1), (2), and (3):

Bα + αC→Bα^T^ + αC^T^ (4)

It means that an extended dislocation is transmitted across CTB and cross-slips in the twin. With increasing applied strain, this extended dislocation continually cross-slips and is transmitted across all of the CTBs.

By above dislocation reaction, an extended dislocation propagates across the NT nanowires along the zigzag slip plane, which leaves a step on the surface but nothing within nanowires. Such dislocation reactions cause a relief in flow stress. Similar dislocation reactions continually process to sustain the plastic deformation with the further applied strain.

**Figure 8:**
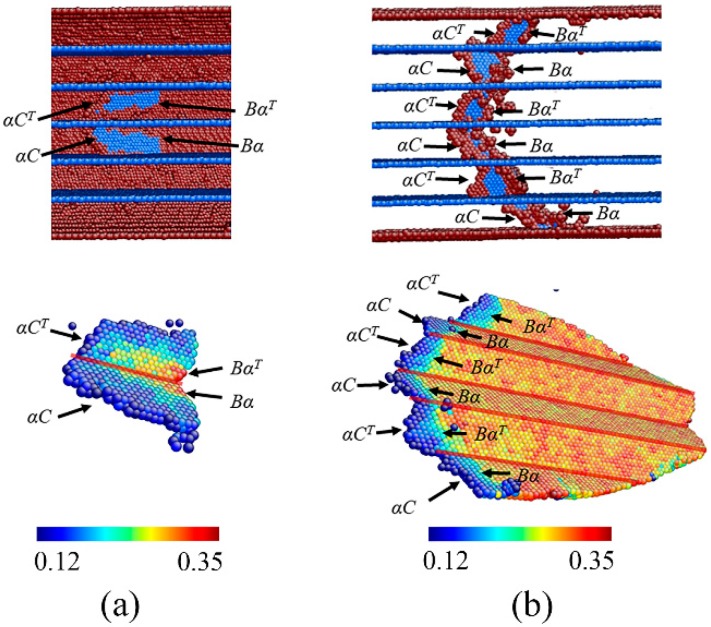
Snapshot of the NT Au nanowire with θ = 90° and λ = 2.83 nm during stretching at stain of (**a**) 3.33%, (**b**) 3.40%. On the bottom panel, the atoms are colored according to the SVA, where the atoms with SVA less than 0.12 nm are hid. On the top panel, the atoms are colored according to the CNA, where the atoms with FCC lattice are hid and blue and red atoms represent the atoms with HCP lattice and defective atoms. The red lines mark the CTBs.

We also found that twin spacing only plays a minor role in the deformation mechanism. When the twin spacing decreases to 2.83 nm, the restriction of CTBs on the dislocation sliding enhances such that the first leading partial cannot slip across the nanowires without restriction. Following the nucleation, the first leading partial transmits across the CTB by the dislocation reaction to release another partial in the twin:

αC→αC^T^ + α^T^α (5)

where αC^T^ is a partial that can slip away in the twin on the BCD^T^ plane. α^T^α is a stair-rod dislocation. Immediately following this leading partial dislocation, a trailing dislocation is nucleated on the same plane accompanied by the abrupt relief in flow stress. Thus, weakening strain hardening and smooth elastic-plastic transition is found in NT Au nanowires with θ = 90° and with small twin spacing. By this leading-trailing partial dislocation pair, an extended dislocation forms separated by a stacking fault. When this extended dislocation reaches to CTB, it could be constricted to form a perfect dislocation, and it subsequently transmits across the CTB followed by the subsequent dislocation dissociation in the twin through the dislocation reactions (1)–(3), as shown Figure 8a. Repeating this dislocation process, the extended dislocation can easily transmit all of the CTBs. Therefore, a necklace-like zigzag extended dislocation forms in the NT nanowires, as illustrated in Figure 8b. This zigzag extended dislocation consists of multiply extended dislocation, each bounded by two CTBs. The zigzag extended dislocations rapidly and collectively slip across the nanowires along zigzag slip plane with further applied strain, which leaves a step on the surface but nothing within nanowires. Obviously, the collective movement of this zigzag extended dislocations need larger shear stress than that of single extended dislocations, giving rise to high strength. Similar dislocation reactions continually process to sustain the plastic deformation with the increase of applied strain. Similar dislocation structures have been reported in columnar-grained NT Cu [39] and NT Cu nanowires with parallel CTBs [15].

### 3.5. Unique Strain Hardening Mechanism of the NT Nanowires with θ = 54.74°

**Figure 9:**
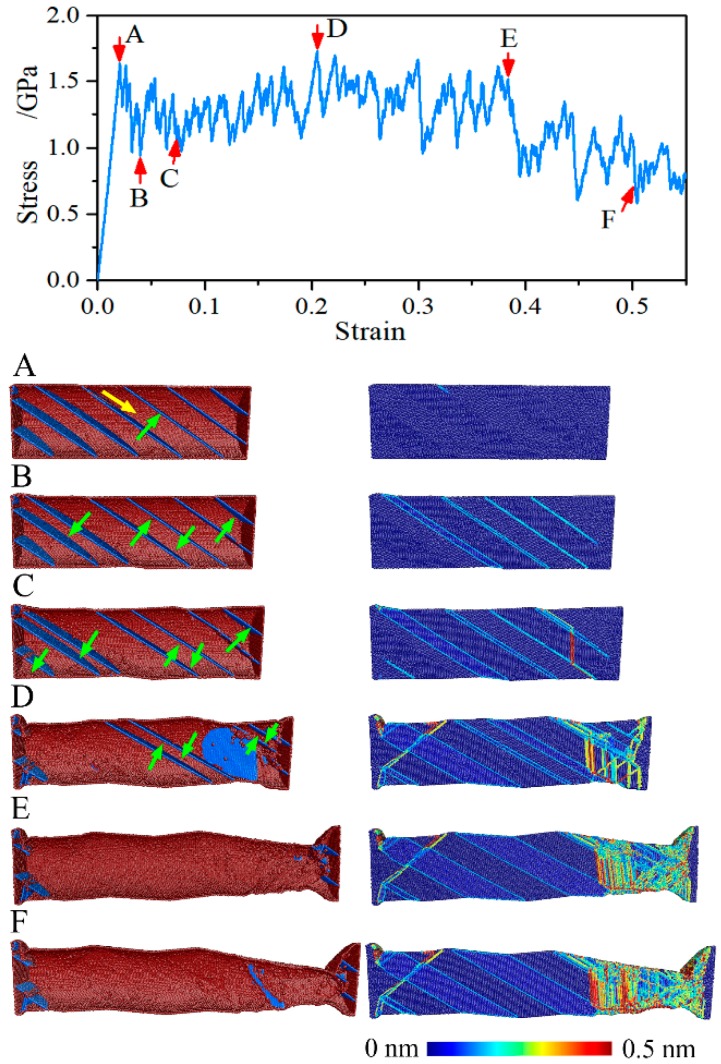
Tensile stress-strain curves and snapshot corresponding to labeled points of the NT Au nanowire with θ = 54.74° and λ = 2.83 nm. On the left panel, the atoms are colored according to the CNA, where the atoms with FCC lattice are hid. On the right panel, the atoms are colored according to the SVA, where the atoms outside of the thresholds are visible and are drawn in saturated colors. Yellow and green arrows denote the direction of dislocations slide and CTBs migration, respectively.

A unique strain hardening behavior is observed in the tensile deformation of the NT Au nanowires with θ = 54.74°, which is related to its particular feature of tensile deformation. Figure 9 captures the atomic structures of the NT nanowire with θ = 54.47° and λ = 5.45 nm corresponding to labeled points in its stress-strain curve. The emission of a twinning partial from the CTB-surface intersection site on the free surface is responsible for the initial yielding at the strain of 2.08% (point A). This twin dislocation slips unrestrictedly along the CTB until it passes across the nanowire, which results in the migration of the CTB (point B). With the increase of applied strain, the Twin migration continuously develops and extends to almost every CTB, as illustrated in Figure 9 at point C and Point D. The Twin migration stops when two neighboring CTBs meet and turns into a stacking fault. With the further applied strain, the stacking fault annihilates by the subsequent trailing partial dislocation. The above-mentioned dislocation processes repeat and repeat until almost all of the CTBs annihilate at the strain of 38.55% (point E). The initial NT nanowire thus transforms into a defect-free single crystal nanowire, although small twin segments still present at the both end of the nanowire as the artificially frozen atoms. The Twin migration mediated plastic deformation is also found in in-situ test [13,40] and MD simulation [29,31] of NT Cu nanowires with slanted CTBs.

When the strain is less than 38.55% (point E), Twin migration is the governing mode of plastic deformation although some leading partial and subsequent trailing partial are activated at the moving end of the nanowire, as shown in Figure 9. When the strain increases over 38.55%, the slide of leading partial and subsequent trailing partial fully sustains the plastic deformation, as illustrated in Figure 9 at point F, which is consistent with the single crystal Au nanowires, and the stress drops until the failure of the nanowire. It is noted that the stress has inconspicuous reduction in the strain range of 0 to 38.55%, and the ultimate stress is achieved at the strain of 29.82% (point D) but not at the strain of 38.55% (point E) where the NT nanowire turns into a single crystal nanowire. The NT Au nanowire with θ = 54.74° and with λ = 1.41 nm and 5.45 nm have similar deformation mechanism. Like the macroscale tensile deformation, the plastic deformation of the NT Au nanowires with θ = 54.74° at strain hardening stage is homogeneous deformation but with different deformation mechanism. The subsequent strain softening means the occurrence of deformation localization.

The energy barrier for the twinning partial nucleation is lower than the partial nucleation because the CTB provided additional energy for partial nucleation, which means that the nucleation of the twining partial need smaller stress than partials dislocation. Hence, Twin migration controlled plastic deformation is responsible for the unique strain hardening in NT Au nanowires with θ = 54.74°. The energy difference between nucleation of twinning partial and partial dislocation, which can be coarsely equivalent to the twin energy, can be linked to the hardening capability, i.e., the larger twin energy, the larger hardening capability. Therefore, the present NT Au nanowires show smaller strain hardening factor than the NT Cu nanowires as its smaller twin energy [29].

**Figure 10:**
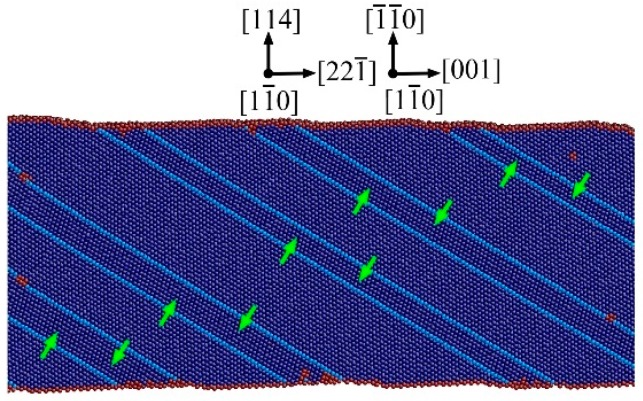
Twin migration of the NT Au nanowire with θ = 54.74° and λ = 2.83 nm at the strain of 0.12. The atoms are colored according to the CNA. The green arrows denote the direction of CTBs migration.

We also found that the CTBs migration always results into the growth of the grains with [001] axis accompanied by the disappearance of the grains with [22$\bar{1}$] axis for the NT Au nanowires with θ = 54.74°, regardless of twin spacing, as shown in Figure 10. Thus, the resulted single crystal Au nanowires has [001] orientation. Moreover, all the leading partials and subsequent trailing partials nucleate and slip in the grain with a [001] axis. Such observation suggests that grain with [001] axis is preferred upon tensile loading. The reorientation through Twin migration has generally reported in single crystalline FCC nanowires with very small lateral dimensions (below 5 nm), accompanied by a shape memory effect [41,42]. Here, we have shown that this Twin migration also led to a unique hardening behavior in NT Au nanowires with θ = 54.74°, but without shape memory effect.

### 3.6. Deformation Mechanism of the NT Au Nanowires with θ = 19.47°

**Figure 11:**
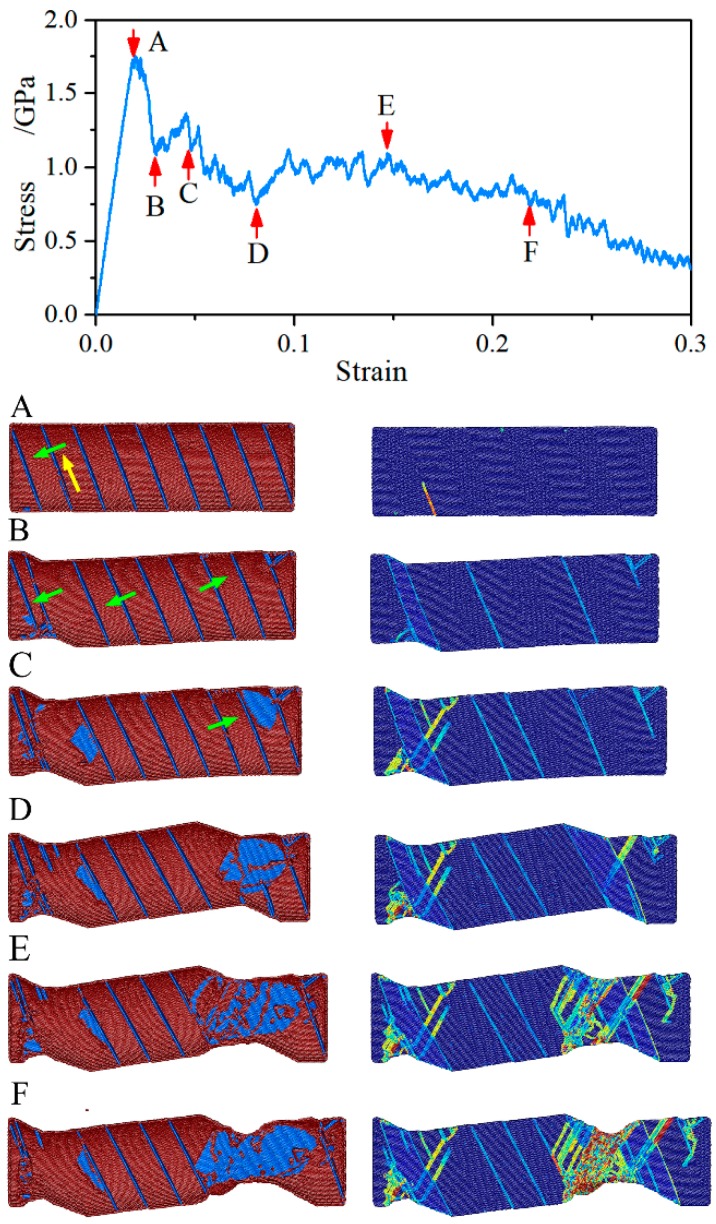
Tensile stress-strain curve and snapshot corresponding to labeled points of the NT Au nanowire with θ = 19.47° and λ = 2.83 nm. On the left panel, the atoms are colored according to the CNA, where the atoms with FCC lattice are hid. On the right panel, the atoms are colored according to the SVA, where the atoms outside of the thresholds are visible and are drawn in saturated colors. Yellow and green arrows denote the direction of dislocations slide and CTBs migration, respectively.

A typical example of atomic-scale deformation in the NT Au nanowires with θ = 54.74° and with λ = 5.45 nm is represented in Figure 11. We have shown that the NT Au nanowires with θ = 19.47° have the same yield mechanism to those with θ = 54.74°, i.e., emission of a twinning partial (point A). Interesting, the NT Au nanowires with θ = 19.47° show strain softening other than unique strain hardening that is found in Au nanowires with θ = 54.74°. With the increase of the applied strain, the Twin migration is observed (point B), which is majorly responsible for the first stress drop. Still, we noted that just two CTBs remarkably migrate (point C and point D), and only one CTB can unrestrictedly slip until it approaches its neighboring CTB and is annihilated by the subsequent trailing partial (point D). Hence, the activation of partials on the surface and subsequent slide along the slip plane intersected with CTB mainly sustain the plastic deform of the NT Au nanowires with θ = 19.47°. Notably, the plastic deformation concentrates on the region where CTBs migrate away and multiple bursts of dislocation activations on different slip systems are observed (point E and point F). Moreover, the obvious torsion of sample can be found, which is a result of the migration of CTBs. The above atomic-scale analysis shows that strain softening in NT Au nanowires with θ = 19.47° is related to partial dislocation sliding controlled plastic deformation, which is just the plastic deformation mechanism for the single crystal nanowires with FCC lattice where the strain softening was observed [5,19].

### 3.7. Twin Orientation, Slip System and Dislocation Process

The above observation clearly shows that the active slip system and dislocation process are strongly dependent on the twin orientation in the NT Au nanowires, which resulted into drastically different mechanical behavior and strain hardening behavior, as summarized in Table 2. This observation is analyzed in the following by a Schmid’s factor analysis to predict active slip systems.

The maximum Schmid’s factors of {111}<112> slip systems in matrix and twinned grain were calculated and are listed in Table 3. When orientation angle is 0° and 90° showing orthogonally and parallel CTBs, the Schmid’s factor of the twinning partial is zero such that Twin migration is suppressed. As generally reported, slip-twin interaction is responsible for the plastic deformation, which also leads to a general strain hardening behavior in NT nanowires with orientation angle of 0° [23,33]. The repeated slip-twin interaction destroys the original growth CTBs. In contrast, slip transmission is observed in NT nanowires with an orientation angle of 90°, where the multiple cross slip of extend dislocation into the twin forms a specific a necklace-like zigzag extended dislocation. Notably, this slip transmission cannot destroy the initial growth CTBs.

When orientation angle is changed to 19.47° showing slant CTBs, the Schmid’s factor of the twinning partial reaches to 0.31, which is still smaller than that of the partial dislocation. Our simulations indicated that the twinning partial was activated ahead of the partial dislocation with large Schmid’s factor in the NT Au nanowires with θ = 19.47°. This is caused by the low dislocation active energy of the CTB-surface intersection site where the first twinning partial emits. As the large Schmid’s factor, partial dislocations continually nucleate and propagate subsequently sustaining major plastic deformation. With the increase of twin orientation, the resolved shear stress on CTBs becomes large, which is favorable to the nucleation of the twinning partial. When twin orientation is increased to 54.74°, the twinning partial has equal Schmid’s factor to partial dislocation in matrix grain, and even more large in twin grain. Thus, twin migration becomes the dominating deformation mechanism until almost all the CTBs disappear. Similar transformation of the deformation mechanism related to the twin orientation is found in the plate-like NT Cu sample [33], in agreement with our simulations. Above all, as twin orientation increases from 0° to 90°, the dominating deformation mechanism shifts from slip-CTB interaction to dislocation slip, Twin migration, and slip transmission in sequence.

## 4. Conclusions

In conclusion, larger scale molecular dynamics simulations were performed to elucidate the effect of twin orientation and spacing on mechanical behavior, especially the strain hardening and deformation mechanism at the atomic level of the cylindrical NT Au nanowires. The main conclusions were drawn as follows:

(1) With the twin orientation varying from 0° to 90°, two types of strain hardening behaviors and a strain softening behavior are found. A general strain hardening behavior in the initial stage of plastic deformation is observed with twin orientation of 0° and 90°, which resulted by the blockage effect of twin boundary on the initial dislocation sliding. A unique strain hardening behavior at a larger strain (0.21 to 0.31) is rebuilt when twin orientation is changed to 54.74°, which is related to twin migration. Because of this unique strain hardening, an improved ductility is achieved.
(2) The twin orientation plays a main role in the mechanical behavior and deformation mechanism of the cylindrical NT Au nanowires. The orthogonally oriented CTBs have the optimal strengthening capability in the NT Au nanowires, followed by the parallel CTBs and the slant CTBs.
(3) As twin orientation increases from 0° to 90°, the dominating deformation mechanism shifts from slip-CTB interaction to dislocation slip, Twin migration, and slip transmission in sequence.

## Figures and Tables

**Table 1:** The lattice orientations of load axis for the specific twin orientation.

	0°	19.47°	54.74°	90°
**Matrix**	[111]	[112]	[001]	[11$\bar{2}$]
**Twin**	[111]	[552]	[22$\bar{1}$]	[$\bar{1}\bar{1}$2]

**Table 2:** The resulted mechanical behavior, first active dislocation and nucleation site, and dominated deformation mechanism, depending on the twin orientation angle.

	Mechanical Behavior	First Active Dislocation	Nucleation Site of the First Dislocation	Dominated Deformation Mechanism
**0°**	General hardening	Partial	CTB-surface intersection site	Slip-twin interaction
**19.47°**	Softening	Twinning partial	CTB-surface intersection site	Slip
**54.74°**	Unique hardening	Twinning partial	CTB-surface intersection site	Twin migration
**90°**	General hardening	Partial	Surface between adjacent CTBs	Slip transmission (zigzag extended dislocation)

**Table 3:** Maximum Schmid factors of twinning partial (TP) and partial (P) under different twin orientation angle.

	0°		19.47°		54.74°		90°	
	TP	P	TP	P	TP	P	TP	P
**Matrix**	0	0.31	0.31	0.39	0.47	0.47	0	0.39
**Twin**	0	0.31	0.31	0.49	0.47	0.26	0	0.39
